# Podiatric assessment in children and adults with epidermolysis bullosa: are foot and ankle patient-reported outcome measures (PROMs) able to measure the severity of podiatric involvement among EB patients?

**DOI:** 10.1097/JW9.0000000000000046

**Published:** 2023-01-19

**Authors:** Joy Lynn Wong, Mohammed Tariq Khan, Timothy L. Cowan, Dedée F. Murrell

**Affiliations:** a Faculty of Medicine, University of New South Wales, High Street, Kensington NSW, Sydney, Australia; b Department of Dermatology, St George Hospital, Gray Street, Kogarah NSW, Sydney, Australia; c Premier Specialists, Dermatology Trials Australia, Kogarah, NSW, Australia; d The Sydney Children’s Hospitals Network, High Street, Randwick NSW, Sydney, Australia; e Department of Epidermolysis Bullosa, Great Ormond Street Hospital for Sick Children’s, London, UK; f Faculty of Health Sciences and Wellbeing, University of Sunderland, Sunderland, UK; g Department of Podiatric Medicine and Orthopaedics, Temple University, Philadelphia, USA

**Keywords:** blistering skin, epidermolysis bullosa, foot care, patient-reported outcome measure, podiatry, rare disease

## Abstract

Epidermolysis bullosa (EB) represents a group of rare genetic skin fragility disorders characterized by (muco) cutaneous blistering upon minimal mechanical trauma. Ninety percent of EB patients experience podiatric symptoms which may affect physical functioning and emotional well-being. To date, an EB-specific podiatric assessment has not been outlined to guide clinicians in the assessment of EB podiatric involvement. This review describes the podiatric involvement of patients with EB and assesses the relevance of validated foot and ankle patient-reported outcome measures (PROMs) in measuring podiatric severity among EB patients. A literature review was conducted to identify systematic reviews and clinical studies investigating foot health and podiatric manifestations using validated foot health PROMs across foot and ankle conditions. Limited studies have documented the significance of podiatric involvement among EB patients. Existing EB-specific PROMs are not region-specific for assessing podiatric involvement. Among the foot and ankle PROMs, the Foot Health Status Questionnaire, Foot Function Index, and Manchester Oxford Foot Questionnaire were identified as potentially appropriate for assessing podiatric severity among EB patients, each with its strengths and limitations in assessment. However, they have not been widely validated for assessing dermatology-related diseases. An evaluation of the relevance of each identified PROM to EB podiatric assessment would enable future development of an appropriate EB-specific podiatric assessment tool that would guide management.

**What is known about this subject in regards to women and their families?**

- Epidermolysis bullosa (EB) disproportionately affects children, with a greater incidence in younger age groups, as seen in a cohort study across 17 EB centers.
- The majority of those with EB experience podiatric symptoms and blistering of the foot, which negatively affect physical functioning, appearance, and self-esteem, especially in young people and women.
- To date, no EB-specific podiatric assessment tool has been developed to objectively assess patient-reported outcomes.

**What is new from this article as messages for women and their families?**

- Identification of existing validated foot patient-reported outcome measures used in other podiatric-related conditions and how they could be adapted for use in EB assessment across all demographics and age groups.
- Explore assessment domains relevant to EB patient-reported outcomes including foot function, activity limitations, pain, footwear, and foot health-related quality of life.

## Introduction

This review will discuss the podiatric involvement of epidermolysis bullosa (EB) patients and assess the relevance of validated patient-reported outcome measures (PROMs) to foot problems in patients with EB. The purpose of this study was to explore the suitability of commonly used foot and ankle PROMs and potentially adapt them for use in studies assessing podiatric severity and interventions among EB patients.

## Methods

A literature review was conducted to identify clinical studies and systematic reviews investigating foot health and podiatric manifestations using validated foot health PROMs across foot and ankle conditions. The most recent articles relating to podiatric assessment were identified across electronic search engines: National Institutes of Health databases (PubMed and Medline), Google Scholar, and the Consensus-based Standards for the Selection of Health Measurement Instrument (COSMIN) database of systematic reviews. Key phrases used to identify such articles include “Foot [Abnormalities, Analysis, Drug Effects, Injuries, Pathology, Surgery],” “foot deformities/foot diseases/foot ulcer,” “Surveys and Questionnaires,” “patient outcome assessment/treatment outcome/quality improvement/quality indicators.”

## Overview of EB and literature documenting podiatric severity

### Introduction to EB

EB represents a group of rare inherited skin fragility disorders characterized by painful blistering of the skin upon minor mechanical trauma, leading to erosions and non-healing ulcers.^3^ EB typically presents at birth or early in life with an incidence of approximately 20 per million live births.^4^ EB disproportionately affects children; most EB patients fall under the 1 to 9 and 10 to 19 age groups, according to a cohort study across 17 EB centers.^5^

There are 4 main groups of EB (EB Simplex, Junctional EB, Dystrophic EB, Kindler EB), classified according to the level of skin cleavage. They are further classified based on the mode of inheritance and a combination of phenotypic, ultrastructural, immunohistochemical, and molecular findings.^6^ The distribution and severity of skin blistering are dependent on the subtype of EB—blistering in milder forms is confined to the hands and feet, while severe forms of EB can lead to extensive chronic wounds that cause significant disability.^3^

### Podiatric involvement among EB patients and its significance

**Fig. 1.**
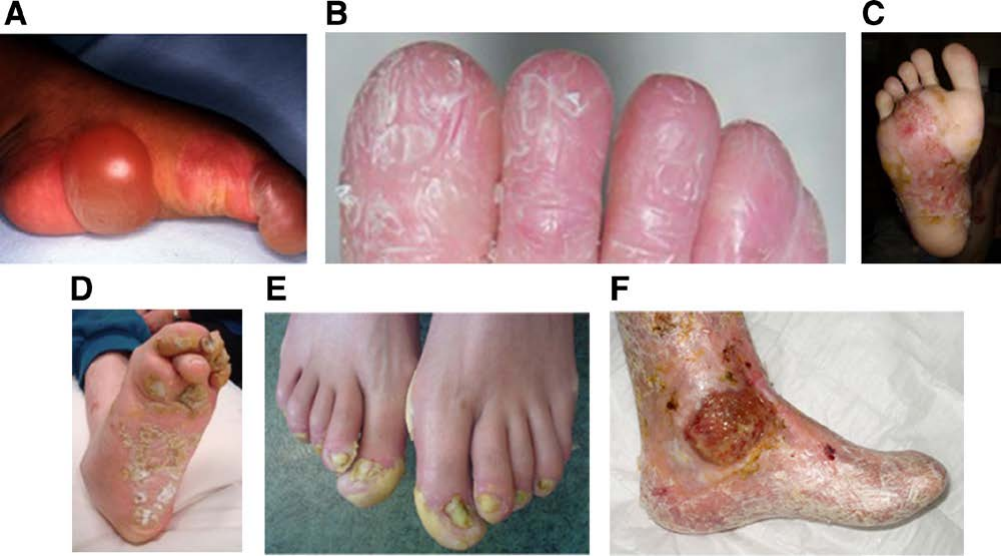
Podiatric manifestations in epidermolysis bullosa (EB) patients (Courtesy of Professor Dédée F. Murrell and Dr. Mohammed Tariq Khan, Sydney, Australia). (A) Vesicle formation and blistering of the foot. (B) Loss of toe nails (anonychia) in EB simplex. (C) Erosions and scarring of the foot in junctional EB. (D and E) Keratoderma of the foot and nails in EB simplex. (F) Fusion of toes (pseudosyndactyly).

Ninety percent of patients with EB have podiatric manifestations, including blistering, hyperkeratosis, nail dystrophy, and structural abnormalities affecting foot positioning.^7,8^ Figure 1A–F illustrates some of the podiatric manifestations found in patients with EB. Foot pain in EB is often neuropathic and sometimes unbearable.^9^ These podiatric manifestations inadvertently affect physical well-being and activities of daily living. A cohort study reflected a significant percentage of EB patients experiencing walking or standing limitations due to cutaneous pain, with only up to one-third of all children with EB being independent in walking.^10^ In more severe types of EB, foot deformities such as extension and flexion contractures of the toes, equinus, cavus, and pseudosyndactyly have been observed, resulting in permanent reliance on a wheelchair.^11^

Such podiatric manifestations can exacerbate psychological difficulties and self-esteem. In a study interviewing children with EB, concerns about appearance, isolation, and feelings about being different were raised.^12^ Studies have shown that restricted choices of therapeutic and functional footwear can lead to feelings of exclusion, self-consciousness, and vulnerability.^13^ This is especially significant among female patients, where an emotional relationship and sense of personal identity tied to the type of shoe worn may be negatively impacted by podiatric manifestations.^14^

Despite the impact of podiatric involvement among patients with EB, limited literature has documented its significance. Less priority may be given to addressing foot problems among EB patients when other medical concerns demand more urgent attention.^15^ Recently, Khan et al. published an evidence-based guideline on foot care in EB and noted the importance of validated foot assessment tools to measure the foot health status of EB patients.^7^

### EB studies assessing podiatric interventions

Among the few studies assessing podiatric interventions used among EB patients, none used a validated foot assessment tool to measure patient outcomes.^11,16–18^ Instead, subjective methods of observation or numerical pain scales were used to assess the efficacy of the intervention. Their subjective methods of patient outcome assessment undermine the clinical relevance of the intervention introduced. Hence, a validated EB-specific foot PROM would standardize the parameters of measured patient improvement from the intervention.

### EB assessment tools

EB patient assessment tools have been developed, and questionnaires such as QOLEB, BEBS, EBDASI, and iscorEB have been validated and are reliable in distinguishing subtypes of EB.^19–24^ However, these tools often offer comprehensive rather than region-specific assessment approaches. For example, iscorEB evaluates skin, mucosal, and internal organ involvement, laboratory abnormalities, and complications.^19^ As such, these disease-specific assessment tools may not be applicable to podiatric/region-specific interventions and consequently downplay the severity of foot involvement compared to other regions affected.

### Psychometric properties

Minimal clinically important difference (MCID) is defined as the smallest difference in score in the domain of interest which patients perceive as beneficial and which would mandate, in the absence of troublesome side effects and excessive cost, a change in the patient’s management.^23^

Cronbach α is the measure of internal consistency: how closely related a set of items are as a group. Cronbach α ≥ 0.70 for each unidimensional scale or subscale was sufficient.^23^

Intraclass correlation coefficient (ICC) is a widely used reliability index in test–retest reliability analyses.^24^

## Assessment of foot and ankle PROMs

### Importance of PROMs

PROMs can be categorized as generic health status measures, disease-specific health status measures, and anatomic scales. Most foot-related PROMs developed are anatomic scales which are region-specific to the foot and/or ankle.^25^ They are used to objectively detect changes in a patient’s foot health status in response to an intervention and assist clinicians’ understanding of the effects of podiatric manifestations on one’s functioning.^26,27^

Despite the severity of podiatric involvement and its impact on foot health-related quality of life (HRQoL) among patients with EB, no research has been undertaken to determine the suitability of foot PROMs for genetic dermatoses involving the feet.

### PROMs used across foot and ankle conditions

A 2017 systematic review identified 50 PROMs available across all foot and ankle conditions, the majority of which were anatomic-type instruments.^28^ As shown in Table 1, none of the identified PROMs has been consistently used across all foot and ankle conditions. Moreover, some PROMs were favored in their use for specific categories of foot and ankle pathologies based on the content properties of its questions, and multiple PROMs were usually used together to assess podiatric outcomes.

**Table 1:** Systematic and literature reviews evaluating patient-reported outcome measures (PROMs) across various foot and ankle conditions

Year	Author	Study type	Condition	Purpose of study	Top PROMs	Type of PROM	Comment
2017	Jia et al.^28^	Systematic review	All foot and ankle conditions	(1) Identify currently available PROMs for foot or ankle disease patients (2) Critically appraise, compare and synthesize the psychometric evidence for the identified PROMs	FFI MOXFQ	Anatomic Anatomic	Across 50 instruments for patients with foot or ankle diseases, the FFI was the most explored instrument and the MOXFQ had the best overall evidence for psychometric properties
2018	Sierevelt et al.^39^	Systematic review	Orthopedic foot and ankle conditions	To identify the most frequently used foot and ankle-specific PROMs in recent orthopedic foot and ankle literature	FFI FOAS FAAM	Anatomic Anatomic Anatomic	The 3 most commonly used outcome measures were the FFI, FAOS, FAAM, capturing 50% of the citations of all 21 outcome measures. Evidence for some psychometric properties of the FAOS and FAAM are lacking/limited
2019	Ortega-Avila et al.^56^	Systematic review	Rheumatoid arthritis	To identify self-reported outcome measures specific to the foot and ankle in patients with rheumatoid arthritis and to investigate the methodological quality and psychometric properties of these measures	SEFAS FHSQ FAAM SAFE	Anatomic Anatomic Anatomic Disease	SEFAS has the best overall psychometric properties and methodological quality, and is currently the most appropriate PROM available for patients with rheumatoid arthritis
2018	Lundgren-Nilsson et al.^57^	Systematic review	Osteoarthritis	To describe the available PROMs used in osteoarthritis and their performance quality	WOMAC SF36 KOOS	Disease Generic Disease	WOMAC, SF36 and KOOS were the most frequently used PROMs in osteoarthritis published papers from 2000 to 2016
2015	Schrier et al.^58^	Literature review	Hallux Surgery	To identify and rate available PROMs in hallux valgus surgery	MOXFQ SEFAS VAS SF36	Anatomic Anatomic Generic Generic	The MOXFQ scores best on positively rated qualities, and SEFAS is a less ideal but good alternative. The VAS is the best pain score and the SF36 the best general health assessment tool
2019	Ortega-Avila et al.	Systematic review	Diabetic foot	To identify PROMs that are specific to diabetic foot and evaluate their psychometric properties and methodological quality	FHSQ FAAM	Anatomic Anatomic	Among the 11 PROMs identified, the FHSQ provided the best overall psychometric properties, while FAAM is the most commonly used PROM for diabetic foot studies
2012	Hogg et al.^59^	Systematic review	Diabetic foot	To summarize and analyze the literature pertaining to HRQoL PROMs used in the spectrum of diabetes-related foot disease	CWIS SF36 DFUS NeuroQoL	Generic Generic Disease Disease	Of the generic tools, the SF-36 shows sensitivity to foot disease and has been used most frequently. Of the disease-specific tools, the DFUS and NeuroQoL are the most validated. The CWIS shows promise in assessing HRQOL in active ulceration, but is non-specific for diabetic foot ulcers
2014	Gorecki et al.^60^	Systematic review	Chronic wounds; pressure ulcer (PU)	To identify generic, PU-specific and chronic skin wound-specific PROMs used to assess HRQoL in patients with PUs or other chronic skin wounds and determine their suitability for use in patients with PUs	SF-36	Generic	Findings demonstrate that each PROM presented some degree of irrelevant content for this population. The most frequently applied measure used in PU HRQoL research, the SF36, is not conceptually comprehensive in its assessment of HRQoL outcomes important in PUs

CWIS, Cardiff Wound Impact Schedule; FAAM, Foot and Ankle Ability Measure; FFI, Foot Function Index; FAOS, Foot and Ankle Outcome Score; HRQOL, health-related quality of life; KOOS, Knee Injury and Osteoarthritis Outcome Score; MOXFQ, Manchester Oxford Foot Questionnaire; SAFE, Salford Rheumatoid Arthritis Foot Evaluation; SEFAS, Self-Reported Foot and Ankle Score; SF-36, 36 Item Short Form Survey (QoL); VAS, Visual Analogue Scale (pain); WOMAC, Western Ontario and McMaster Universities Osteoarthritis Index.

For orthopedic conditions, anatomic foot PROMs (Foot Health Status Questionnaire [FHSQ], Manchester Oxford Foot Questionnaire [MOXFQ], Foot Function Index [FFI], Foot and Ankle Outcome Score [FAOS], Foot and Ankle Ability Measure [FAAM], Self-Reported Foot and Ankle Score [SEFAS]) have been favored in clinical studies. Osteoarthritis-related clinical studies favor disease-specific tools (Western Ontario and McMaster Universities Osteoarthritis Index, Knee Injury and Osteoarthritis Outcome Score, FAOS). For diabetic foot, a mixture of anatomic (FHSQ, FAAM), disease-specific (DFUS), and generic (Cardiff Wound Impact Schedule [CWIS], SF36) PROMs has been favored in clinical studies. In clinical studies related to chronic wounds, a generic PROM (SF36) was used to comprehensively assess HRQoL outcomes.

**Fig. 2.**
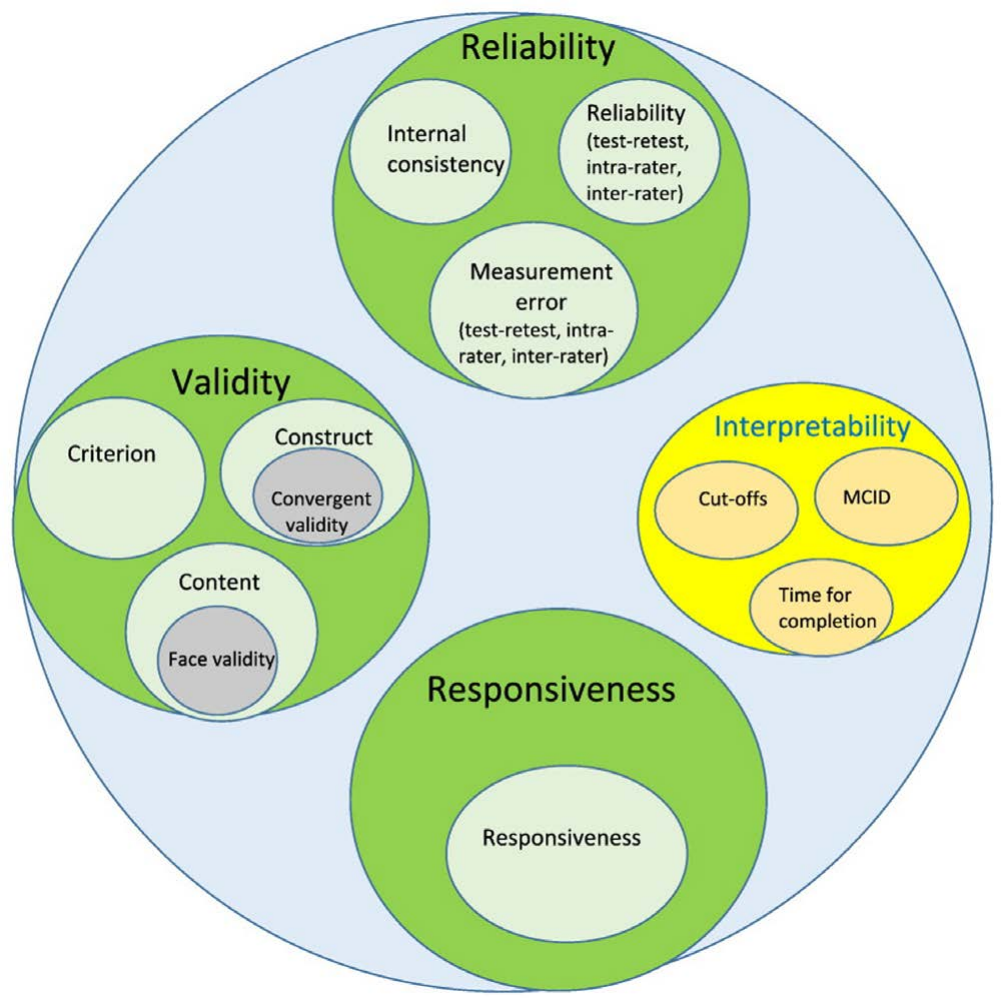
Consensus-based Standards for the Selection of Health Measurement Instruments (COSMIN) taxonomy of psychometric properties to validate a patient-reported outcome measures (PROM).^1^

The wide spectrum of podiatric involvement among patients with EB makes it difficult to choose a single suitable PROM to measure the effect of podiatric interventions. The questionnaire’s purpose and content, practical considerations (administration, interpretability, etc.), and evidence for psychometric properties (Fig. 2) using the COSMIN should be evaluated to determine suitability.^1^

Table 2 describes the domains and properties of PROMs (FHSQ, FFI, MOXFQ, FAAM, FAOS, SEFAS, DFUS, CWIS) relevant to assessing EB-specific foot manifestations. Of the tools evaluated in Table 2, 3 region-specific instruments (FHSQ, FFI, MOXFQ) were deemed potentially appropriate for EB podiatric studies based on their purpose, intended population, and content of the questions. Evidence for the psychometric properties of the FHSQ, FFI, and MOXFQ is compiled in Table 3.

**Table 2:** Description of self-administered patient-reported outcome measures (PROMs)

PROM	Author	Year	No. of items and domains	Purpose	Domains	Feasibility	Response options	Scoring interpretation
Foot Health Status Questionnaire (FHSQ)	Benett et al.^29^	1998	13 items; 4 domains	Measure foot HRQoL	• Pain • Function • Footwear • General foot health	3–5 min	5-point Likert scale 1 = None 5 = Severe/always	Individual item scores are re-coded, tabulated, and transformed to a scale ranging from 0 to 100 for each of the 4 domains Higher scores represent better foot health
Foot Function Index (FFI)	Budiman-Mak et al.^37^	1991	23 items; 3 domains	Measure impact of foot pathology on function	• Pain • Disability • Activity limitations	3–5 min	VAS 1 = No pain/no difficulty 10 = Worst pain imaginable Unable	The VAS for each item is measured to obtain a score out of 10. The item scores are totaled and then divided by the maximum total possible for all of the subscale items which the patient indicated were applicable Higher scores represent worse foot health
Manchester Oxford Foot Questionnaire (MOXFQ)	Dawson et al.^47^	2006	16 items; 3 domains	PROM for surgery of the foot and ankle	• Activity limitation (walking/standing) • Pain • Social interaction	3–5 min	5-point Likert scale 0 = None 4 = Severe/all of the time	Scores for each domain are calculated by summing the responses to each item within a given domain. Raw scores can be converted to a 0–100 metric Higher scores represent worse foot health
Foot Ankle Outcome Score (FAOS)	Roos et al.^55^	2001	42 items; 5 domains	Adapted from the KOOS—to evaluate symptoms and functional limitations related to the foot and ankle	• Pain • Other Symptoms (Stiffness Swelling, and Range of Motion) • Activities of Daily Living • Sport and Recreational Activities • Foot and Ankle-Related Quality of Life	5–10 min	5-point Likert scale 0 = None/never 4 = Extreme/always	Each of the 5 subscale scores is calculated as the sum of the items included. A normalized score ranging from 1 to 100 is calculated for each subscale Higher scores represent worse foot health
Foot and Ankle Ability Measure (FAAM)	Martin et al.^61^	2005	29 items; 2 domains	Assess physical performance among individuals with a range of leg, foot, and ankle musculoskeletal disorders	• Activities of Daily Living • Sports	5–0 min	5-point Likert scale 0 = Unable to do 4 = No difficulty	Each item on the subscale is scored from 0 to 4. N/A responses are not counted. The score on each of the items are added together and the total score is divided by the highest potential score, which is then transformed into a percentage. Subscale scores range from 0 to 100 Higher scores represent better foot health

HRQOL, health-related quality of life; KOOS, Knee Injury and Osteoarthritis Outcome Score; VAS, Visual Analogue Scale (pain).

**Table 3:**
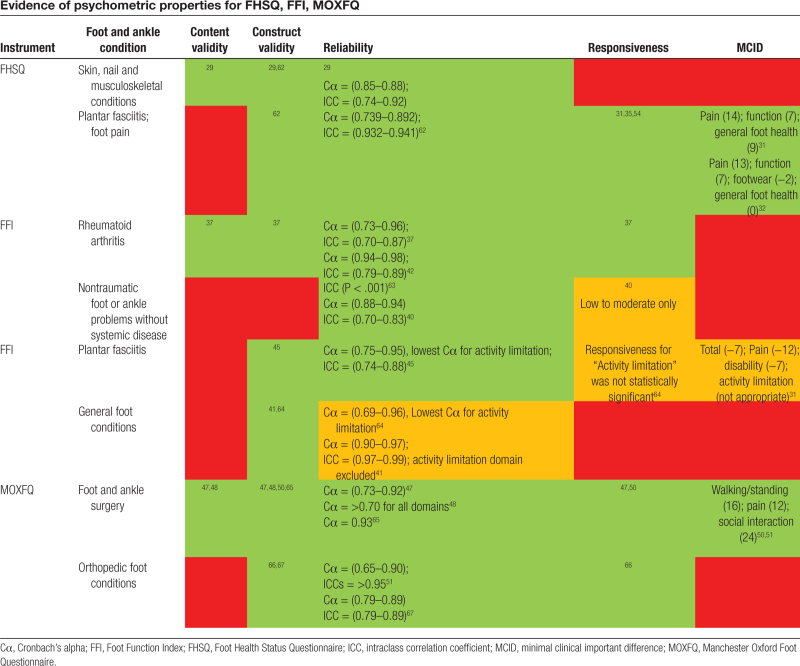
Evidence of psychometric properties for FHSQ, FFI, MOXFQ

## Foot Health Status Questionnaire

The FHSQ (Supplementary Material A, http://links.lww.com/IJWD/A10) is a region-specific instrument developed in 1998 by Bennett et al. intended for the assessment of patients undergoing surgical treatment for common foot conditions. The FHSQ is a 13-item questionnaire with 4 domains: foot pain, foot function, footwear, and general foot health. Its purpose is to measure foot HRQoL.^29^

### Strengths

The FHSQ is a short questionnaire that is easily administered and assesses domains relevant to EB podiatric patients. It has been used extensively across a variety of foot and ankle conditions, and has established reasonable psychometric properties. In a developmental study involving 101 participants with skin, nail, and musculoskeletal conditions of the foot, a high degree of internal consistency (Cronbach α = 0.85–0.88), test-retest reliability (intraclass correlation coefficient range = 0.74–0.92), and validity (content, construct, and criterion) were determined.^29^ In a 2019 systematic review identifying 11 PROMs specific for diabetic foot, the FHSQ provided the best overall psychometric properties, illustrating its usefulness in nonorthopedic-related foot and ankle conditions.^30^

In studies assessing customized foot orthoses in patients with foot pain and plantar fasciitis, responsiveness across all 4 subscales was observed, and MCID was determined.^31,32^ This suggests favorable use of the FHSQ in measuring patient outcomes among patients with EB receiving conservative podiatric interventions.

### Limitations

The quality of studies evaluating the psychometric properties of the FHSQ was questioned, which noted inconsistencies in the FHSQ development process and potential limitations in its use.^28,33^ A practical disadvantage would be the tediousness of score interpretation using a designated program which requires respondents to answer all questions or risk having all their data discarded.^33^ Some items within the subscales overlapped, creating redundancy, for example, “How would you rate your overall foot health? and “In general, what condition would you say your feet are in?.”^33^ Additionally, the FHSQ may be unsuitable for children, where 50% of the items may be incomprehensible by respondents aged below 11.^34^ This increases the respondent burden because a significant proportion of patients with EB are children.

The results of a trial evaluating foot orthoses in plantar fasciitis suggest that the FHSQ may be more responsive to foot conditions without marked disability.^35^ Additionally, in a prospective cohort study of 115 adults with musculoskeletal foot pain, people with multimorbidity scored worse in each domain of the FHSQ both before and after podiatric intervention than those without.^36^ Both studies suggest that individuals with multimorbidity and marked disability, such as more severe EB forms, are less likely to perceive modest improvements in foot health.

## Foot function index

The original FFI (Supplementary Material B, http://links.lww.com/IJWD/A11) is the most explored region-specific instrument across all foot and ankle conditions, developed by Budiman-Mak et al. in 1991.^28^ It consists of 23 items across 3 domains: pain, disability, and activity limitations, with the aim of quantifying the impact of foot pathology.^37^ Since its development, the FFI has been revised and modified, but its revised versions have not been extensively utilized and validated.

### Strengths

One of the main advantages of the FFI is its extensive usage across various foot and ankle pathologies, including congenital, acute and chronic diseases, injuries, and surgical corrections.^38^ The FFI has also been used in studies involving children, adults, and elderly populations.^38^ FFI usage, therefore, appeals to clinical studies involving the EB population, where a wide age range exists.

The FFI was developed with good reliability and validity. In the developmental study assessing 87 subjects with rheumatoid arthritis, the FFI demonstrated high internal consistency (Cronbach α = 0.73–0.96), reliability (intraclass correlation coefficient = 0.70–0.87 during 1 week), and construct validity.^37^ Responsiveness and MCID values were also demonstrated in studies measuring the outcomes of patients with plantar fasciitis.^31,35^

### Limitations

The methodological quality of studies involving the FFI is insufficient to determine the quality of its psychometric properties. Poor evidence of the psychometric properties of the FFI has been demonstrated by 2 systematic reviews evaluating foot- and ankle-specific PROMs.^28,39^

The content of the original FFI was considered unsuitable for many patient populations across clinical studies owing to the floor effects of items from the domain of “activity limitations.” Questions regarding the “use of assistive devices indoors/outdoors,” and “stay in bed/indoors most of the day because of foot problems,” were selected as non-applicable by many patients across several studies, particularly those without marked disability.^40–46^ As such, the FFI appears to be more suitable for low-functioning patients and should be modified according to the study population characteristics.^35,39^

A practical limitation is the time-consuming efforts of administration, coding, and data entry of the visual analogue scale (pain) score. However, this could be circumvented by using a Likert 5-point scale instead, such as the validated Dutch FFI-5 point version.^40^

Despite the limitations of the FFI, studies that have modified the FFI (by removing non-applicable questions) and its scoring properties (conversion to a Likert point scale) have shown that it remains a reasonable instrument. Similarly, the FFI can be modified according to the EB population studied.

## Manchester Oxford Foot Questionnaire

MOXFQ (Supplementary Material C, http://links.lww.com/IJWD/A12) is a PROM for surgery of the foot and ankle and was developed in 2006 using the MFPDQ as a template.^47^ This 16-item questionnaire covers 3 domains: functional limitation (walking/standing), pain, and social interaction. It uses a 5-point Likert scale for scoring, and a summary index score is calculated.

### Strengths

One of the key strengths of the MOXFQ is its extensive psychometric evaluation as a foot and ankle surgery PROM, which has provided the best evidence for its psychometric properties across 50 instruments identified for foot and ankle pathology.^28^ It is well validated in its use across a variety of foot and ankle surgical studies, according to a study which confirmed its suitability among patients undergoing foot and ankle surgery at UK hospitals, with responses showing no floor/ceiling effects.^48^

In addition to the responsiveness detected in the original validation sample of hallux valgus patients,^47^ it also appeared to be sensitive to changes 6 months after elective foot surgery.^49^ The MCID estimates for each subscale have also been determined in various studies.^50,51^ The MOXFQ is a succinct questionnaire that is easy to interpret and complete and has a high item response rate.^34,52^

### Limitations

The use of the MOXFQ in nonsurgical studies has been limited and has not been thoroughly examined. Items are more specific to measuring surgical outcomes, which may not be applicable to the majority of EB patients with milder subtypes, thereby creating a floor effect. Moreover, assessment of footwear difficulties is limited to only 1 item enquiring about self-consciousness regarding the type of shoes worn, which may not reflect broader footwear issues faced by patients.^53^ Additionally, the MOXFQ is not as responsive to change for conservative podiatric interventions such as foot orthoses, as seen in a study comparing the MOXFQ against the FHSQ.^54^

## Other PROMs

Similar to MOXFQ, FAOS and FAAM are region-specific PROMs used in podiatric surgical studies. Additionally, evidence for some psychometric properties (measurement error, content, and structural validity) of the FAOS and FAAM was lacking according to a systematic review.^39^ The extensive list of items of the FAOS and FAAM mainly focuses on the functional limitations of the foot/ankle (Supplementary Materials D and E, http://links.lww.com/IJWD/A13). Questions of the FAOS placed greater emphasis on joint mobility and symptoms specific to osteoarthritis, which may not be as relevant to milder subtypes of EB.^55^ However, certain items from the FAOS and FAAM which were not covered by the FHSQ, FFI, and MOXFQ should be considered for their relevance. This includes questions pertaining to foot/ankle joint symptoms, sports and recreational activities, and foot HRQoL.

The domains assessed (social activities, emotions, etc.) in the DFUS and CWIS largely overlapped with those from the validated QOLEB tool; hence, they were not considered for further analysis. Similarly, SEFAS was excluded from consideration, as it only consisted of 12 items covered by the FHSQ, FFI, MOXFQ, FAOS, and FAAM.

## Conclusion

To date, no study has formally assessed the effectiveness of podiatric interventions in patients with EB by using a validated foot and ankle PROM. Although validated EB-specific instruments are available, they are not region-specific. The FHSQ, FFI, and MOXFQ were deemed potentially suitable for EB podiatric assessment—they were easy to administer, demonstrated good psychometric properties, and assessed domains relevant to EB patients. Each PROM has its strengths and limitations in use. The FHSQ covered a good range of domains, including footwear, but may not be suitable for assessing EB patients with marked disability and comorbidities. The FFI is the most extensively used tool across various foot and ankle conditions, but some items are not applicable to those without significant disability or assistive devices. The MOXFQ demonstrated the best psychometric properties but has not been widely used in nonsurgical studies; therefore, it is limited in its use among EB patients with milder subtypes (the majority). FAAM and FAOS should also be considered for their wide range of questions, some of which are applicable to EB patients. Such insights into the suitability of foot and ankle PROMs for measuring podiatric severity among EB patients can be used to develop an appropriate EB-specific podiatric PROM, which will set objective parameters for measuring podiatric outcomes and enable clinicians to improve podiatric management among EB patients.

## Conflicts of interest

None.

## Funding

None.

## Study approval

N/A.

## Author contributions

JW, TK, and DM contributed to research design, performance of research, data analysis, and writing of the paper. TC contributed to performance of research and writing of the paper.

## Acknowledgments

We wish to thank the following developers of identified foot health questionnaires: Dr Paul Bennett (FHSQ), Dr Elly Budiman-Mak (FFI), Dr Jill Dawson (MOXFQ), Professor Martin Robroy (FAAM), Professor Ewa M. Roos (FAOS). We also wish to thank the clinicians and staff at the EB/bullous clinics of St. George Hospital, Sydney, and Royal Melbourne Hospital. Finally, we would like to thank UNSW librarian, Ressie Davis, for her expert advice on the development of the literature review search strategy.

## Supplementary Material
