# Supplementary material for: Podiatric assessment in children and adults with epidermolysis bullosa: are foot and ankle patient-reported outcome measures (PROMs) able to measure the severity of podiatric involvement among EB patients?
Source: Int J Womens Dermatol. 2023 Jan 19;9(1):e046. doi: 10.1097/JW9.0000000000000046 (PMC9851710; doi:10.1097/JW9.0000000000000046)
Supplement: Supplementary file 1 [file jw9-9-e046-s001.pdf]

The Foot Health Status Questionnaire

INSTRUCTIONS

- This questionnaire asks for your views about your foot health.
- All you need to do is circle your answer to each question.
- If you are unsure about how to answer a question, please give the best answer you can.

The following questions are about the foot pain you have had during the past week.

1. What level of foot pain have you had during the past week ?

(circle number)

None……………………………………1

Very Mild……………………………… 2

Mild…………………………………… 3

Moderate…………………………….. 4

Severe………………………………… 5

(circle a number for each question below)

**DURING THE LAST WEEK...**	Never	Occasionally	Fairly Many Times	Very Often	Always
**2.** How often have you had foot pain ?	1	2	3	4	5
**3.** How often did your feet ache?	1	2	3	4	5
**4.** How often did you get sharp pains in your feet ?	1	2	3	4	5

These questions are about how much your feet interfere with activities you might do during a typical day.

(circle a number for each question below)

DURING THE LAST WEEK…….

	Not at All	Slightly	Moderately	Quite a bit	Extremely
**5.** Have your feet caused you to have difficulties in your work or activities ?	1	2	3	4	5
**6.** Were you limited in the kind of work you could do because of your feet ?	1	2	3	4	5

DURING THE LAST WEEK…

	Not at All	Slightly	Moderately	Quite a bit	Extremely
**7.** How much does your foot health limit you walking ?	1	2	3	4	5
**8.** How much does your foot health limit you climbing stairs ?	1	2	3	4	5

9. How would you rate your overall foot health ? (circle number)

Excellent…………………………. 1
Very Good……………………….. 2
Good……………………………..… 3
Fair……………………………….. 4
Poor………………………………. 5

Please turn to the next page

The following questions are about the shoes that you wear. Please circle the response which best describes your situation.

		Strongly Agree	Agree	Neither Agree nor Disagree	Disagree	Strongly Disagree
**10.**	It is hard to find shoes that do not hurt my feet.	1	2	3	4	5
**11.**	I have difficulty in finding shoes that fit my feet.	1	2	3	4	5
**12.**	I am limited in the number of shoes I can wear.	1	2	3	4	5

13. In general, what condition would you say your feet are in ?

(circle number)

Excellent……………………… 1

Very Good…………………… 2

Good………………………….. 3

Fair……………………………. 4

Poor…………………………… 5

Please write some comments about the current state of your feet:

……………………………………………………………………………………

……………………………………………………………………………………

……………………………………………………………………………………

……………………………………………………………………………………

……………………………………………………………………………………

Supplemental Material A. Foot Health Status Questionnaire (Foot health section)
