# Supplementary material for: Podiatric assessment in children and adults with epidermolysis bullosa: are foot and ankle patient-reported outcome measures (PROMs) able to measure the severity of podiatric involvement among EB patients?
Source: Int J Womens Dermatol. 2023 Jan 19;9(1):e046. doi: 10.1097/JW9.0000000000000046 (PMC9851710; doi:10.1097/JW9.0000000000000046)
Supplement: Supplementary file 2 [file jw9-9-e046-s002.pdf]

Supplemental Material B. Foot Function Index

DATE: ________________

NAME ___________________________________ SURNAME _____________________________________

This questionnaire has been designed to give your therapist information as to how your foot pain has affected your ability to manage in everyday life. Please answer every question. For each of the following questions, we would like you to score each question on a scale from 0 (no pain or difficulty) to 10 (worst pain imaginable or so difficult it required help) that best describes your foot over the past WEEK.

Pain Subscale: How severe is your foot pain:

1. **Foot pain at its worst?** No pain ________________ Worst Pain Immaginable
2. **Foot pain in morning?** No pain ________________ Worst Pain Immaginable
3. **Pain walking barefoot?** No pain ________________ Worst Pain Immaginable
4. **Pain standing barefoot?** No pain ________________ Worst Pain Immaginable
5. **Pain walking with shoes?** No pain ________________ Worst Pain Immaginable
6. **Pain standing with shoes?** No pain ________________ Worst Pain Immaginable
7. **Pain walking with orthotics?** No pain ________________ Worst Pain Immaginable
8. **Pain standing with orthotics?** No pain ________________ Worst Pain Immaginable
9. **Foot pain at end of day?** No pain ________________ Worst Pain Immaginable

Disability Subscale: How much difficulty did you have:

10. **Difficulty walking in house?** No difficulty ________________ So difficult unable
11. **Difficulty walking outside?** No difficulty ________________ So difficult unable
12. **Difficulty walking 4 blocks?** No difficulty ________________ So difficult unable
13. **Difficulty climbing stairs?** No difficulty ________________ So difficult unable
14. **Difficulty descending stairs?** No difficulty ________________ So difficult unable
15. **Difficulty standing tip toe?** No difficulty ________________ So difficult unable
16. **Difficulty getting up from chair?** No difficulty ________________ So difficult unable
17. **Difficulty climbing curbs?** No difficulty ________________ So difficult unable
18. **Difficulty walking fast?** No difficulty ________________ So difficult unable

Activity Limitation Subscale: How much of the time do you:

19. **Stay inside all day because of feet?** None of the time ________________ All of the time
20. **Stay in bed because of feet?** None of the time ________________ All of the time
21. **Limit activities because of feet?** None of the time ________________ All of the time
22. **Use assistive device indoors?** None of the time ________________ All of the time
23. **Use assistive device outdoors?** None of the time ________________ All of the time

Score: ____/230 points x 100= ___%
