# Supplementary material for: Podiatric assessment in children and adults with epidermolysis bullosa: are foot and ankle patient-reported outcome measures (PROMs) able to measure the severity of podiatric involvement among EB patients?
Source: Int J Womens Dermatol. 2023 Jan 19;9(1):e046. doi: 10.1097/JW9.0000000000000046 (PMC9851710; doi:10.1097/JW9.0000000000000046)
Supplement: Supplementary file 3 [file jw9-9-e046-s003.pdf]

MANCHESTER-OXFORD FOOT QUESTIONNAIRE (MOXFQ)

Circle as appropriate:<br>**RIGHT / LEFT**<br>***<u>During the past 4 weeks</u> this has applied to me:***	*Please tick ✓ one box for each statement*				
	**None of the time**	**Rarely**	**Some of the time**	**Most of the time**	**All of the time**
**1.** I have pain in my foot/ankle	❑	❑	❑	❑	❑
**2.** I avoid walking long distances because of pain in my foot/ankle	❑	❑	❑	❑	❑
**3.** I change the way I walk due to pain in my foot/ankle	❑	❑	❑	❑	❑
**4.** I walk slowly because of pain in my foot/ankle	❑	❑	❑	❑	❑
**5.** I have to stop and rest my foot/ankle because of pain	❑	❑	❑	❑	❑
**6.** I avoid some hard or rough surfaces because of pain in my foot/ankle	❑	❑	❑	❑	❑
**7.** I avoid standing for a long time because of pain in my foot/ankle	❑	❑	❑	❑	❑
**8.** I catch the bus or use the car instead of walking, because of pain in my foot/ankle	❑	❑	❑	❑	❑
**9.** I feel self-conscious about my foot/ankle	❑	❑	❑	❑	❑
**10.** I feel self-conscious about the shoes I have to wear	❑	❑	❑	❑	❑

Please turn to next page

***During the past 4 weeks** this has applied to me:*	*Please tick ✓ one box for each statement*				
	**None of the time**	**Rarely**	**Some of the time**	**Most of the time**	**All of the time**
**11.** The pain in my foot/ankle is more painful in the evening	❑	❑	❑	❑	❑
**12.** I get shooting pains in my foot/ankle	❑	❑	❑	❑	❑
**13.** The pain in my foot/ankle prevents me from carrying out my work/everyday activities	❑	❑	❑	❑	❑
**14.** I am unable to do all my social or recreational activities because of pain in my foot/ankle	❑	❑	❑	❑	❑

15. During the past 4 weeks how would you describe the pain you usually have in your foot/ankle? *(please tick one box)*

None	Very mild	Mild	Moderate	Severe
❑	❑	❑	❑	❑

16. During the past 4 weeks have you been troubled by pain from your foot/ankle in bed at night? *(please tick one box)*

No nights	Only 1 or 2 nights	Some nights	Most nights	Every night
❑	❑	❑	❑	❑

Supplemental Material C. Manchester-Oxford Foot Questionnaire

Finally, please check that you have answered every question

Thank you very much

©MOXFQ v2 University of Oxford, Department of Public Health (HSRU), Old Road Campus, Oxford OX37LF, UK
