# Supplementary material for: Podiatric assessment in children and adults with epidermolysis bullosa: are foot and ankle patient-reported outcome measures (PROMs) able to measure the severity of podiatric involvement among EB patients?
Source: Int J Womens Dermatol. 2023 Jan 19;9(1):e046. doi: 10.1097/JW9.0000000000000046 (PMC9851710; doi:10.1097/JW9.0000000000000046)
Supplement: Supplementary file 4 [file jw9-9-e046-s004.pdf]

FAOS FOOT & ANKLE SURVEY

Todays date: _____/______/______ Date of birth: _____/______/______

Name: ____

INSTRUCTIONS: This survey asks for your view about your foot/ankle. This information will help us keep track of how you feel about your foot/ankle and how well you are able to do your usual activities.
Answer every question by ticking the appropriate box, only one box for each question. If you are unsure about how to answer a question, please give the best answer you can.

Symptoms

These questions should be answered thinking of your foot/ankle symptoms during the **last week**.

S1. Do you have swelling in your foot/ankle?

Never	Rarely	Sometimes	Often	Always
☐	☐	☐	☐	☐

S2. Do you feel grinding, hear clicking or any other type of noise when your foot/ankle moves?

Never	Rarely	Sometimes	Often	Always
☐	☐	☐	☐	☐

S3. Does your foot/ankle catch or hang up when moving?

Never	Rarely	Sometimes	Often	Always
☐	☐	☐	☐	☐

S4. Can you straighten your foot/ankle fully?

Always	Often	Sometimes	Rarely	Never
☐	☐	☐	☐	☐

S5. Can you bend your foot/ankle fully?

Always	Often	Sometimes	Rarely	Never
☐	☐	☐	☐	☐

Stiffness

The following questions concern the amount of joint stiffness you have experienced during the **last week** in your foot/ankle. Stiffness is a sensation of restriction or slowness in the ease with which you move your joints.

S6. How severe is your foot/ankle stiffness after first wakening in the morning?

None	Mild	Moderate	Severe	Extreme
☐	☐	☐	☐	☐

S7. How severe is your foot/ankle stiffness after sitting, lying or resting **later in the day**?

None	Mild	Moderate	Severe	Extreme
☐	☐	☐	☐	☐

Pain

P1. How often do you experience foot/ankle pain?

Never	Monthly	Weekly	Daily	Always
☐	☐	☐	☐	☐

What amount of foot/ankle pain have you experienced the **last week** during the following activities?

P2. Twisting/pivoting on your foot/ankle

None	Mild	Moderate	Severe	Extreme
☐	☐	☐	☐	☐

P3. Straightening foot/ankle fully

None	Mild	Moderate	Severe	Extreme
☐	☐	☐	☐	☐

P4. Bending foot/ankle fully

None	Mild	Moderate	Severe	Extreme
☐	☐	☐	☐	☐

P5. Walking on flat surface

None	Mild	Moderate	Severe	Extreme
☐	☐	☐	☐	☐

P6. Going up or down stairs

None	Mild	Moderate	Severe	Extreme
☐	☐	☐	☐	☐

P7. At night while in bed

None	Mild	Moderate	Severe	Extreme
☐	☐	☐	☐	☐

P8. Sitting or lying

None	Mild	Moderate	Severe	Extreme
☐	☐	☐	☐	☐

P9. Standing upright

None	Mild	Moderate	Severe	Extreme
☐	☐	☐	☐	☐

Function, daily living

The following questions concern your physical function. By this we mean your ability to move around and to look after yourself. For each of the following activities please indicate the degree of difficulty you have experienced in the **last week** due to your foot/ankle.

A1. Descending stairs

None	Mild	Moderate	Severe	Extreme
☐	☐	☐	☐	☐

A2. Ascending stairs

None	Mild	Moderate	Severe	Extreme
☐	☐	☐	☐	☐

For each of the following activities please indicate the degree of difficulty you have experienced in the **last week** due to your foot/ankle.

A3. Rising from sitting

None	Mild	Moderate	Severe	Extreme
☐	☐	☐	☐	☐

A4. Standing

None	Mild	Moderate	Severe	Extreme
☐	☐	☐	☐	☐

A5. Bending to floor/pick up an object

None	Mild	Moderate	Severe	Extreme
☐	☐	☐	☐	☐

A6. Walking on flat surface

None	Mild	Moderate	Severe	Extreme
☐	☐	☐	☐	☐

A7. Getting in/out of car

None	Mild	Moderate	Severe	Extreme
☐	☐	☐	☐	☐

A8. Going shopping

None	Mild	Moderate	Severe	Extreme
☐	☐	☐	☐	☐

A9. Putting on socks/stockings

None	Mild	Moderate	Severe	Extreme
☐	☐	☐	☐	☐

A10. Rising from bed

None	Mild	Moderate	Severe	Extreme
☐	☐	☐	☐	☐

A11. Taking off socks/stockings

None	Mild	Moderate	Severe	Extreme
☐	☐	☐	☐	☐

A12. Lying in bed (turning over, maintaining foot/ankle position)

None	Mild	Moderate	Severe	Extreme
☐	☐	☐	☐	☐

A13. Getting in/out of bath

None	Mild	Moderate	Severe	Extreme
☐	☐	☐	☐	☐

A14. Sitting

None	Mild	Moderate	Severe	Extreme
☐	☐	☐	☐	☐

A15. Getting on/off toilet

None	Mild	Moderate	Severe	Extreme
☐	☐	☐	☐	☐

For each of the following activities please indicate the degree of difficulty you have experienced in the **last week** due to your foot/ankle.

A16. Heavy domestic duties (moving heavy boxes, scrubbing floors, etc)

None	Mild	Moderate	Severe	Extreme
☐	☐	☐	☐	☐

A17. Light domestic duties (cooking, dusting, etc)

None	Mild	Moderate	Severe	Extreme
☐	☐	☐	☐	☐

Function, sports and recreational activities

The following questions concern your physical function when being active on a higher level. The questions should be answered thinking of what degree of difficulty you have experienced during the **last week** due to your foot/ankle.

SP1. Squatting

None	Mild	Moderate	Severe	Extreme
☐	☐	☐	☐	☐

SP2. Running

None	Mild	Moderate	Severe	Extreme
☐	☐	☐	☐	☐

SP3. Jumping

None	Mild	Moderate	Severe	Extreme
☐	☐	☐	☐	☐

SP4. Twisting/pivoting on your injured foot/ankle

None	Mild	Moderate	Severe	Extreme
☐	☐	☐	☐	☐

SP5. Kneeling

None	Mild	Moderate	Severe	Extreme
☐	☐	☐	☐	☐

Quality of Life

Q1. How often are you aware of your foot/ankle problem?

Never	Monthly	Weekly	Daily	Constantly
☐	☐	☐	☐	☐

Q2. Have you modified your life style to avoid potentially damaging activities to your foot/ankle?

Not at all	Mildly	Moderatly	Severely	Totally
☐	☐	☐	☐	☐

Q3. How much are you troubled with lack of confidence in your foot/ankle?

Not at all	Mildly	Moderately	Severely	Extremely
☐	☐	☐	☐	☐

Q4. In general, how much difficulty do you have with your foot/ankle?

None	Mild	Moderate	Severe	Extreme
☐	☐	☐	☐	☐

Thank you very much for completing all the questions in this questionnaire.

Foot and Ankle Ability Measure (FAAM)
Activities of Daily Living Subscale

Please Answer **every question** with **one response** that most closely describes your condition within the past week.
If the activity in question is limited by something other than your foot or ankle mark "Not Applicable" (N/A).

	No Difficulty	Slight Difficulty	Moderate Difficulty	Extreme Difficulty	Unable to do	N/A
Standing	☐	☐	☐	☐	☐	☐
Walking on even Ground	☐	☐	☐	☐	☐	☐
Walking on even ground without shoes	☐	☐	☐	☐	☐	☐
Walking up hills	☐	☐	☐	☐	☐	☐
Walking down hills	☐	☐	☐	☐	☐	☐
Going up stairs	☐	☐	☐	☐	☐	☐
Going down stairs	☐	☐	☐	☐	☐	☐
Walking on uneven ground	☐	☐	☐	☐	☐	☐
Stepping up and down curbs	☐	☐	☐	☐	☐	☐
Squatting	☐	☐	☐	☐	☐	☐
Coming up on your toes	☐	☐	☐	☐	☐	☐
Walking initially	☐	☐	☐	☐	☐	☐
Walking 5 minutes or less	☐	☐	☐	☐	☐	☐
Walking approximately 10 minutes	☐	☐	☐	☐	☐	☐
Walking 15 minutes or greater	☐	☐	☐	☐	☐	☐

Foot and Ankle Ability Measure (FAAM)
Activities of Daily Living Subscale
Page 2

Because of your foot and ankle how much difficulty do you have with:

	No Difficulty at all	Slight Difficulty	Moderate Difficulty	Extreme Difficulty	Unable to do	N/A
Home responsibilities	☐	☐	☐	☐	☐	☐
Activities of daily living	☐	☐	☐	☐	☐	☐
Personal care	☐	☐	☐	☐	☐	☐
Light to moderate work (standing, walking)	☐	☐	☐	☐	☐	☐
Heavy work (push/pulling, climbing, carrying)	☐	☐	☐	☐	☐	☐
Recreational activities	☐	☐	☐	☐	☐	☐

How would you rate your current level of function during you usual activities of daily living from 0 to 100 with 100 being your level of function prior to your foot or ankle problem and 0 being the inability to perform any of your usual daily activities.

__ __ __ . 0 %

Martin, R; Irrgang, J; Burdett, R; Conti, S; VanSwearingen, J: Evidence of Validity for the Foot and Ankle Ability Measure. Foot and Ankle International. Vol.26, No.11: 968-983, 2005.

Foot and Ankle Ability Measure (FAAM)
Sports Subscale

Because of your foot and ankle how much difficulty do you have with:

	No Difficulty at all	Slight Difficulty	Moderate Difficulty	Extreme Difficulty	Unable to do	N/A
Running	□	□	□	□	□	□
Jumping	□	□	□	□	□	□
Landing	□	□	□	□	□	□
Starting and stopping quickly	□	□	□	□	□	□
Cutting/lateral Movements	□	□	□	□	□	□
Ability to perform Activity with your Normal technique	□	□	□	□	□	□
Ability to participate In your desired sport As long as you like	□	□	□	□	□	□

How would you rate your current level of function during your sports related activities from 0 to 100 with 100 being your level of function prior to your foot or ankle problem and 0 being the inability to perform any of your usual daily activities?

__ __ __ . 0%

Overall, how would you rate your current level of function?

□ Normal □ Nearly Normal □ Abnormal □ Severely Abnormal

Martin, R; Irrgang, J; Burdett, R; Conti, S; VanSwearingen, J: Evidence of Validity for the Foot and Ankle Ability Measure. Foot and Ankle International. Vol.26, No.11: 968-983, 2005.
